# Collaborative governance at the start of an integrated community approach: a case study

**DOI:** 10.1186/s12889-022-13354-y

**Published:** 2022-05-19

**Authors:** Sanneke J. M. Grootjans, M. M. N. Stijnen, M. E. A. L. Kroese, D. Ruwaard, M. W. J. Jansen

**Affiliations:** 1 Department of Health Services Research, Faculty of Health, Medicine and Life Sciences, Care and Public Health Research Institute (CAPHRI), Maastricht University, Duboisdomein 30, 6229 GT Maastricht, The Netherlands; 2 Living Lab Public Health Limburg, Public Health Service South Limburg (GGD Zuid Limburg), Het Overloon 2, 6411 TE Heerlen, The Netherlands

**Keywords:** Collaborative governance, Integrated community approach, Case study, Collaboration, Population health management

## Abstract

**Background:**

We studied collaborative governance at the start of an integrated community approach aiming to improve population health, quality of care, controlling health care costs and improving professional work satisfaction. Our objective was to investigate which characteristics of collaborative governance facilitate or hamper collaboration in the starting phase. This question is of growing importance for policymakers and health initiatives, since on a global scale there is a shift towards ‘population health management’ where collaboration between stakeholders is a necessity. In addition, it is crucial to investigate collaborative governance from the beginning, since it offers opportunities for sustainability of collaboration later on in the process.

**Methods:**

We performed a qualitative case study in four deprived neighbourhoods in the city of Maastricht, the Netherlands. An integrated community approach was implemented, involving various stakeholders from the public and private health sectors and provincial and local authorities. Data was collected from December 2016 to December 2018, with a triangulation of methods (50 observations, 24 interviews and 50 document reviews). The Integrative Framework for Collaborative Governance guided data collection and analysis.

**Results:**

We focused on the dynamics within the collaborative governance regime, consisting of principled engagement, shared motivation and capacity for joint action. We found that shared goalsetting, transparency, being physically present, informal meetings, trust and leadership are key aspects at the start of collaborative governance. An extensive accountability structure can both hamper (time-consuming which hinders innovation) and facilitate (keep everybody on board) collaboration.

**Conclusion, brief summary and potential implications:**

The characteristics we found are of significance for policy, practice and research. Policymakers and practitioners can use our lessons learned for implementing similar (population health) initiatives. This case study contributes to the already existing literature on collaborative governance adding to the knowledge gap on the governance of population health approaches.

**Trial registration:**

NTR6543, registration date; 25 July 2017.

## Background

The sustainability of health care is a concern on a global scale due to the ageing population and chronic, complex health complaints [1, 2]. One of the reasons for this complexity is that health is interconnected with many factors outside the realm of the health sector itself [3–5], such as living and working conditions, the work environment, education, the social environment and individual lifestyle factors [6, 7].

In order to address complex health issues and to form sustainable health care systems, population (health) management is introduced as a possible solution [8]. Population health management strives to address the complex health needs of the population at risk and the chronically ill at all points along the health continuum by integrating services across health care, prevention, social care and welfare [9]. Population health management aims to simultaneously improve population health and quality of care, controlling health care costs and improving professional work satisfaction [10]. This requires a collaborative governance structure in which health issues are addressed beyond the health sector, collectively by governmental and non-governmental organisations, rather than independently.

Collaborative governance can be defined as: *‘*the processes and structures of public policy decision making and management that engage people constructively across the boundaries of public agencies, levels of government, and/or the public, private and civic spheres in order to carry out a public purpose that could not otherwise be accomplished’ [11]*.* Although collaborative governance is widely discussed in the literature as a promising approach to solving public issues that cannot be solved by one entity, [11–14] challenges such as power struggles, the risk of misunderstandings, and contradictory goals appear to be frequent and obvious during the collaboration process [15–18].

There are several examples of collaborate governance initiatives aiming to improve health and wellbeing described in the literature. For example, in the United Kingdom, the National Health Service (NHS) states that collaborative clinical networks have been responsible for some significant health improvements [19]. Another example is an area-based program in the Netherlands, where collaborative governance is used as a strategy to tackle health inequalities [20]. However, the evaluation of these kinds of population health initiatives with a collaborative governance structure is often limited to the outcomes related to their formulated goals in terms of health impact [16]. In addition, there is a need for understanding the complexity and context-related factors of collaborative governance in the early stage, as this might affect continuation [21]. Hence, insight into collaborative governance structure and dynamics can help to understand ‘what works and does not work’ in the starting phase of similar initiatives. Therefore, in this paper we used the Integrative Framework for Collaborative Governance of Emerson et al. [11] to study the development and implementation of collaborative governance in an integrated community approach (ICA) in four deprived neighbourhoods in Maastricht, the Netherlands [22]. We chose the framework of Emerson et al. because a central feature of the framework is the collaborative governance regime (CGR), where (complex) patterns of prevailing action, behaviour and dynamics can be analysed, which is of interest in our case and can be of particular interest to policy makers, managers and other health care leaders [23].

### Objectives

The objective of this study was to investigate which characteristics of collaborative governance facilitate or hamper collaboration in the starting phase of an ICA aimed at improving population health and quality of care, controlling health care costs and improving health professional work satisfaction.

## Methods (Aim, Design and Setting)

### Setting

In 2016, an ICA was initiated as part of a pilot project [24] aiming to improve population health and quality of care, control health care costs and improve health professional work satisfaction, also known as the Quadruple Aim [10]. In the ICA, health and social care providers, the municipality, the primary health insurer, the Provincial State, professionals and citizens collaborate within four deprived neighbourhoods in Maastricht, the capital city of the province of Limburg, in the south of the Netherlands. Citizens living in these four neighbourhoods (*n* = 15,290) are socioeconomically deprived compared to the rest of the city of Maastricht (*n* = 122,144): they describe their health less often as ‘good’ (71.5% compared with 79%), have a higher rate of obesity (44% compared to 41%) and have more difficulties making ends meet (30.5% compared with 22%) [25, 26].

### Design

**Fig. 1:**
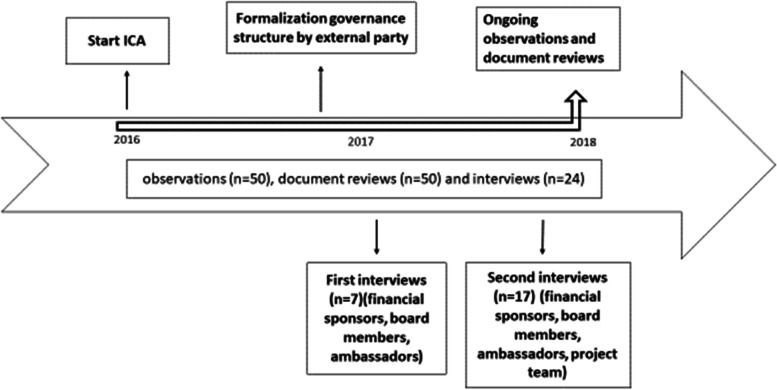
Timeline of the integrated community approach

We performed a qualitative case study into collaborative governance at the start of the ICA. The starting phase of the ICA is defined as the phase in which the collaborative governance network for the ICA is formed and implemented. Fig. 1 shows the timeline of the ICA. We studied the start (December 2016 till December 2018) of the ICA because this phase is intertwined with the viability of the collaboration over a longer period of time [27]. Since the ICA is constantly evolving, we believe a case study is the appropriate way to investigate the objective because it permits a study of the collaborative governance regime in its real-life context [28]. Using formative evaluation, the formation and implementation of the collaborative governance network was investigated using qualitative methods (observations, semi-structured interviews and a document review). An independent researcher conducted the observations, interviews and document review in this case study. Preliminary findings (in themes) were shared with the stakeholders along the continuum of the research period.

### Case study participants

Various stakeholders from the public and private health sectors (including (non-)governmental organisations and private providers) and provincial/local authorities are involved in this case study. Below we elaborate on their role in the ICA.

### Financial sponsors

The financial sponsors fund the ICA and consist of three different players: the dominant health insurer, the Municipality of Maastricht and the Provincial State of Limburg. The role of the financial sponsors is to fund the ICA without having a substantial role in developing the initiatives which are unrolled in the ICA.

### Daily board and independent chair

The daily board consists of five different chief executive officiers (CEOs) of both public and private organisations with one independent chair. The role of the daily board is to make decisions about issues that are discussed in the ambassadors group and to have a close connection to the financial sponsors, project team and ambassadors.

### Ambassadors

The ambassadors consist of 10 CEOs of 10 different organisations in the social and healthcare domains and the health insurer. Some ambassadors are delegates from the daily board. The ambassadors all have the same goal (‘to improve the Quadruple Aim’) and signed a (non-binding) commitment to the intention to place this shared goal above their own organisational goals. The ambassadors’ role is to allow their own employees the freedom to collaborate with other professionals outside their own working domains, and the ambassadors are expected to dissimenate the shared ICA goals among their professionals and employees.

### Project team

The project team consists of five policy advisors delegated by organisations present in both the daily board and the ambassadors group. The role of the project team is to operate and manage the activities which are initiated and to have a connecting role with the citizens and professionals, as well as with the daily board and the ambassadors.

### Citizens and professionals

The ICA utilises a bottom-up approach: this means that the evolution and the implementation of the ICA is tailored to the needs of the community. During the time phase of this case study, the professionals (e.g. general practitioners, social workers and home care nurses) and citizens are represented in the ICA through individual case stories collected by the project team from 2016 onwards.

### External party

**Fig. 2:**
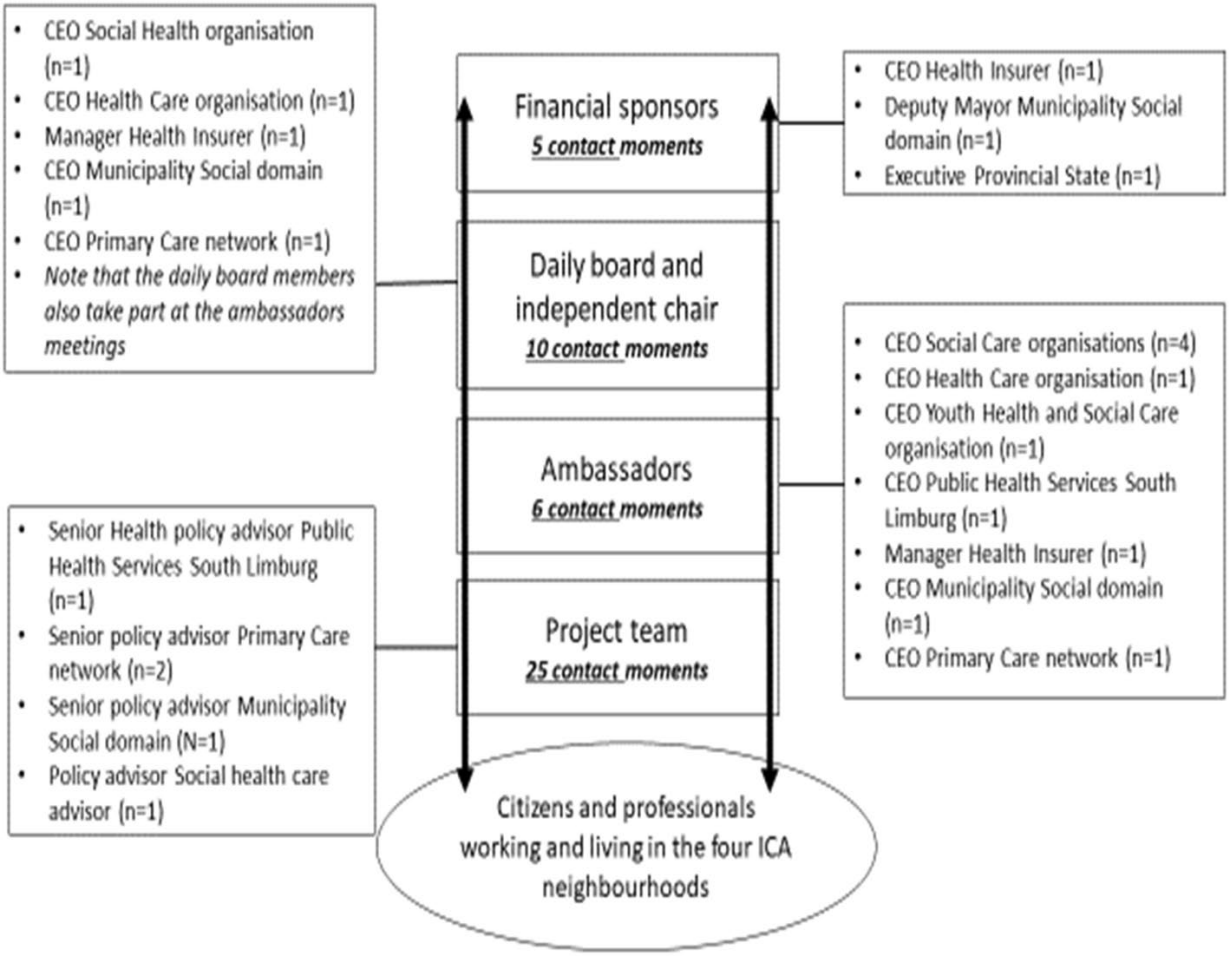
Stakeholders involved in the ICA: December 2016 to December 2018

At the beginning of forming the governance structure, an independent external party was attracted to formalise the governance structure. The external party consisted out of two employees who were expected to give advice regarding the governance structure. Although the party is not an official stakeholder in the ICA, documents and observations where the external party plays a role are included in the data analysis. Fig. 2 shows the stakeholders involved and the contact frequency of the official meetings between the stakeholders.

### Integrated framework for collaborative governance

**Fig. 3:**
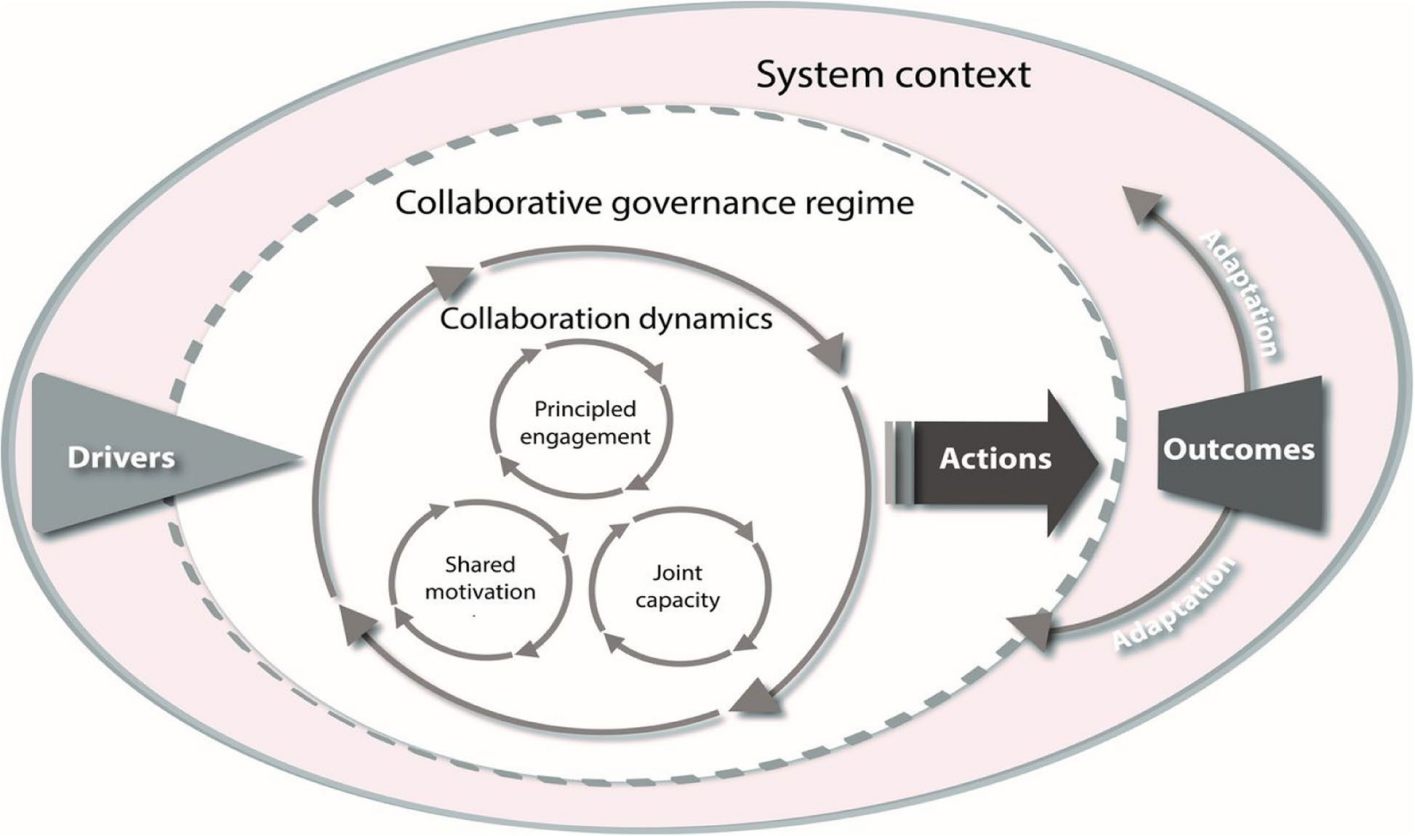
The Integrative framework for collaborative governance. Source: Emerson et al., 2015

In this study, we used the integrated framework for collaborative governance of Emerson et al. [11]. The framework is depicted in Fig. 3.

The framework consists of three nested dimensions: (1) the general system context, (2) the collaborative governance regime (CGR) and (3) collaborative dynamics and collaborative actions. The general system context encompasses the multi-layered context such as socioeconomic, political, legal, environmental and other influences [29, 30]. The general system context has an influence on the CGR and vice versa. Within this context, the framework identifies several ‘drivers’ which are essential for collaboration to unfold. The drivers are leadership (presence of an identified leader), consequential incentives (organisational or external (societal) importance), interdependence (inability to solve a problem on one’s own strength), and uncertainty (shared risk in resolving complex problems) [29]. The CGR is shaped and formed by the drivers but is influenced by ‘collaborative dynamics’. In the concept of CGR, the term ‘regime’ is used to describe the system of public decision making where cross-boundary collaboration is shaped by patterns of behaviour and activity. In the CGR core, three interactive collaborative governance features (principled engagement, shared motivation and capacity for joint action) are visible; they all influence each other and lead to the production of collaborative actions or implementation. Finally, the framework includes ‘impacts’ (the results on the ground) and ‘adaptation’ (the transformation of a complex situation), which will turn into the system context again, as a never-ending cycle, where the ‘drivers’ for collaborative governance will arise again. In this study, we mainly focus on the collaborative dynamics within the CGR since this element of the framework of collaborative governance is fundamental to the starting phase of collaboration.

### Data collection methods

Data were collected with a triangulation of methods. Triangulation increases the knowledge and credibility of qualitative case study research, since it involves the use of multiple methods of data collection about the same phenomenon [30].

### Observations

In order to capture and better understand (group) dynamics, 50 observations were conducted between December 2016 and December 2018 during formal and informal meetings between stakeholders involved in the ICA. These meetings included network meetings, board meetings, ambassador meetings, financial sponsor meetings, project meetings and inter-professional meetings. All observations were stored as commentary field notes to the official meeting minutes distributed to the participants afterwards, filed with the name of the meeting and the date.

### Semi-structured interviews

In order to obtain a detailed insight into the process, we conducted 24 semi-structured interviews with the stakeholders involved. Topics included: stakeholders’ (1) perception regarding last year’s progress in realising shared goals and formation of the collaborative governance network (interviews 2018), (2) attitudes towards and expectations about the collaboration process, (3) views regarding the feasibility of the shared goals and objectives for the ICA, and (4) opinions regarding the extent to which the ICA goals are prioritised above one’s own organisational goals. The interviewer created an open climate to enable participants to present their own beliefs and attitudes and to address additional topics they believed were worth mentioning. Interviews lasted on average 45 to 55 minutes and were audio-recorded and transcribed verbatim.

### Document review

Besides observations and semi-structured interviews, 50 written documents, formal and informal, also formed an important source of collected data. Documents were collected through electronic mail and hard copy papers but also through handouts given at formal presentations. Included in these documents are also official minutes of meetings where the researcher was present as an observer.

### Data analysis

The integrative framework for collaborative governance guided the data analysis. All data were uploaded in NVivo software version 11 and analysed by conventional content coding the text. We used both an inductive and deductive approach to analyse the data. First, the inductive approach was applied while analysing the raw data to discover patterns in the data and to obtain a preliminary set of codes. Subsequently we used a deductive approach using the integrated framework for collaborative governance for grouping the preliminary codes and linking them to the dimensions within the CGR of the integrative framework for collaborative governance. As a codebook we used the structure of the integrative framework with the incorporated nested dimensions and their components as described with examples and definitions by Emerson et al [21]. Incongruence in coding was discussed with a member of the research team (MS). In case of any doubts about a code, the research team (MS, MJ, DR, MK) was asked for their judgement. This iterative process continued until final consensus was reached in the research team about the coding structure. Results of the analysis were also discussed with the stakeholders of the ICA for reliability purposes (member check).

## Results

A broad array of characteristics of collaborative governance were found during data analysis. The key characteristics were found in the collaborative dynamics of the CGR, which consists of principled engagement, shared motivation and capacity for joint action. The results described in this paper are themes of frequent occurrence in both the 50 observations, the 24 interviews, as well as the 50 documents in the ICA. The themes described here came up in at least two out of three data collection methods (observations, interviews and documents) and were mentioned by at least three stakeholders from different organisations involved in the ICA. We will elaborate extensively in the results section on the characteristics found in the CGR.

### Collaborative dynamics: principled Engagement

In the ICA, public and private organisations collaborated across their institutional boundaries. Within their shared ICA goals, the stakeholders also had their own organisational oriented focus. The health insurer had the goal of reducing or equalising healthcare costs, the municipality had the goal of reducing or equalising social care costs, the Provincial State had the goal of increasing the perceived health of the citizens living in the Province of Limburg, and the health organisations had the goal of delivering efficient and quality care. In the middle of 2018, all stakeholders signed a commitment document where they committed to their shared goals.

### (Shared) Goalsetting and transparency

The process of being honest and revealing one’s own organisational goals facilitated the ongoing process, especially at the financial sponsor and the daily board level. At the beginning of the starting phase, tensions arose between stakeholders, which were not directly discussed during official meetings. These tensions mainly arose from stakeholders having financial targets that were very difficult to reconcile with the shared goals of the ICA, i.e. the Quadruple Aim. There was also tension about the possibility of (unequal) shifting of healthcare costs of the insurers towards social care costs of the municipality:

We need to do what is right, but there is a chance when we invest more in people’s (holistic) health awareness that the costs of the municipality will increase, and the healthcare costs of the insurer will decrease…..we need to have a good talk about how we’re dealing with this. Because this means I would invest more money in the project than the health insurer.

(daily board member, interview 2017).

### Physical presence and informal meetings

Being physically present at daily board meetings was important to stimulate transparency and reduce tension. At one of the meetings, a daily board member joined the meeting by conference call. The meeting was in the beginning phase and the board members were still getting to know each other. The physically present board members almost perceived it as a lack of commitment to the ICA by not joining physically (observation, 2018). By ‘defining’ the problem and clashing in the next physical meeting where all members were present, tension was diminished.

There was a lot of tension and distrust between us and some stakeholders, but going through the process of ‘throwing it all on the table’ really made this work for us. I really did not think we would come this far together.

(daily board member, interview 2018).

In addition, observations showed that before the ‘clashing’ occurred in an official meeting, multiple bilateral meetings took place between stakeholders. These meetings also happened ‘un-officially’, i.e. when running into each other at other network meetings in the region, but could also happen in the form of a one-on-one lunch meeting. In this bilateral meeting, the highest tension was often reduced and smoothed out, before the stakeholders discussed the issues in an official ICA meeting later on.

### Collaborative dynamics: shared motivation

Shared motivation is derived from principled engagement, but once in process, shared motivation also influences principled engagement in a cyclical course. In the ICA, building trust between the stakeholders and the investment in their interpersonal relations were a main characteristic we found in interviews and observations.

### Trust

Interpersonal and interorganisational trust grew with the timespan of the ICA. In the beginning, communication and meeting styles were new and needed repeated interactions with each other to reinforce trust.

If I look back at last year, the growth of trust is the primary factor that made us come this far…in the beginning there wasn’t a lot of trust. But getting to know each other by intense collaboration helped to gain trust.

(daily board member, interview 2018).

The bilateral meetings mentioned earlier in principled engagement also contributed to building trust among daily board members, ambassadors and financial sponsors. In the beginning, the project team members had only bilateral meetings with board members of their own organisations. Since there was only communication between the project team and the daily board members outside their mother organisation in official meetings, misunderstandings occurred on a regular basis. For example, evaluation questions by some board members were perceived as critiques on the operational level by the project team, although different organisational communication styles also played a role (e.g. direct vs indirect).

I feel like I’m sometimes misunderstood. Maybe there’s also a difference in communication style. At our company we don’t like to revolve around the issue, we don’t have time for that.

(daily board member, interview 2018).

At the end of the starting phase, the project members had also bilateral meetings with board members outside their own mother organisation. This contributed to the trust building between organisations on multiple levels.

Building trust was also observed in ‘the little things’ during meetings; knowing how somebody likes his/her coffee, knowing the names of his/her kids and making jokes. The members of the ICA started to ‘see’ each other’s interests and issues, which was the foundation of mutual understanding where the stakeholders started to appreciate their different perspectives.

### Collaborative dynamics: capacity for joint action

The essence of capacity for joint action is to generate outcomes through collaboration, which could not be achieved by one stakeholder or organisation on its own. Leadership was a characteristic identified in the observations and interviews as well as in the document review. We identified leadership in multiple roles and both as a driver as well as a characteristic within the CGR. Extensive operating protocols growing in the time span of the ICA was also an identified characteristic.

### Leadership

The ICA was mostly initiated by one person (member of the daily board), who inspired and gathered the main stakeholders involved. In the beginning, this leader arranged and chaired all meetings and had a strong influence on the direction of the ICA. In order to formally create equal support and accountability among the ICA members, an external independent party advised the daily board members to appoint an independent chairman to chair all daily board and ambassadors’ meetings. This independent chair was appointed May 2017. However, the role of the informal leader was still important in stimulating group empowerment and mediation:

I fully support the role of the independent chair, in fact I stimulated it, but do mind I still spent a lot of time having bilateral meetings with stakeholders of the ICA to keep them all aboard. And to be honest, without my input the ICA would run much slower.

(informal leader daily board member, interview 2018).

Also, during the interviews, the other daily board members mentioned strong leadership as a driver of ICA initiation:

I don’t think we would have come this far without this strong vision and drive (from this leader).

(daily board member, interview 2017).

Within the CGR, the informal leader had an overarching role in steering the different projects of the ICA and was in the beginning the linking pin between the daily board and the project team. In the starting phase, the project members where struggling with unrolling the different projects and informing the daily board members about the progress. For example, one of the projects of the ICA was schooling all health and social care professionals in the community with the same holistic vision on health. The informal leader joined the project team meetings several times to guide the team and to function as a mediator between the daily board and the project team (observation, 2018).

### Operating protocols

After the middle of 2017, the ICA started their collaborative actions in order to reach their goals. Starting and implementing the first actions or initiatives and seeing the first (softer) results (e.g. positive feedback from the professionals and citizens about the ICA) gave all stakeholders a boost to their shared motivation:

After all this talking, we finally started with what we are supposed to do, create a sustainable healthcare and social care network.

(ambassador, quote from observation 2018).

However, with the implementation of the ICA, more rules and operating protocols evolved over time creating an extensive accountability structure, especially in terms of evaluating the projects by the project team. For example, one of the daily board members wanted the project team to evaluate the projects with their own organisational key performance indicators (KPI), which were merely focused on financial results and difficult to reconcile with the overarching ICA goals. Also, the balance of operating the protocols and monitoring was a constant discussion among the ICA stakeholders. At one point, in the middle of 2018, the administration of the current projects was perceived as too extensive by the project team:

We don’t have time anymore to actually conduct the current projects and to initiate new projects, since we are only administrating and evaluating…I already work 20 hours extra on top of the formal hours I get for this collaboration*.*

(project team member, interview 2018)

On the other hand, a financial sponsor member stated:

We need more ‘hard results’ to gain trust in the pilot and to keep on sponsoring; a positive process evaluation is not enough to sustain our collaboration in the long run, I need (financially) visible results.

(financial sponsor, interview 2018)

Finding the balance in reporting operating protocols touched on the characteristic of trust, fuelling the dynamics of the CGR to interact with each other:

Why don’t they just trust that we do the right thing? I feel like we have to justify everything we do all the time; this leaves no space for innovation.

(project team member, quote from observation 2018)

## Discussion

In this case study, we used the integrative framework for collaborative governance of Emerson et al. [11] to analyse the start of an ICA. We focused on three interactive components present in the CGR: principled engagement, shared motivation and capacity for joint action. The three components work together to create the foundation of further implementation, collaboration and enrolling of the ICA.

Shared goalsetting and transparency surfaced in several components. The stakeholders formulated Bodenheimer’s Quadruple Aim [10] as their shared goals to address the complex issues they were facing. Having mutually agreed upon goals is discussed in the literature as a crucial condition for successful collaboration [15, 31, 32]. We saw in the ICA that, per stakeholder or organisation, the focus within this shared goal differed. Being open about these differences facilitated collaboration. In addition, being open about own organisational motives touched on the characteristic of trust. Trust both facilitated and hampered the ICA, as was apparent in several dimensions in the CGR (capacity for joint action and shared motivation). With the start of the implementation of the projects in the ICA, the stakeholders received positive feedback from the field, which encouraged their belief and trust in the ICA. In the literature, this is described as ‘intermediate outcomes’ or ‘small wins’ [12, 33, 34]. However, how to reach the overarching goals of the ICA and which of these goals was the most important created tension among the stakeholders. Openly disagreeing and clashing among stakeholders created space for dialogue. Respect and trust among the stakeholders of the ICA were the foundation of this dialogue, where face-to-face contact and time investment was essential.

The facilitative influence of trust in collaborative governance is also broadly discussed in previous research [12, 15, 34]. However, Ran et al. [35] argue that trust in collaborative governance is entangled with ‘power’, where power plays a strong role in creating institutionalised trust [36, 37]. ‘Power’ as a theme itself is not broadly described in the framework of Emerson; nevertheless, it is something which was present in the ICA, coded under the domains of ‘trust’, ‘principled engagement’ and ‘leadership’. French and Raven [18] distinguish five types of power: referent power (power by identification), expert power (power by knowledge attribution), reward power (power by rewarding), coercive power (power by punishment) and legitimate power (power by culture or position). In the ICA, expert and reward power created trust and commitment, since the stakeholders had the feeling that they were committed to the same mission as a team, aiming to improve the Quadruple Aim goals.

With the development of the ICA, the need for operating protocols grew among the stakeholders. Some stakeholders required rules and operating protocols to stay on board of the ICA, since they are dealing with the patterns and accountability culture of their mother organisation. On the other hand, creating these protocols was very time-consuming for the project team members, and little time was left for new projects or even operating the current projects in the ICA. This finding was also found in previous work on collaborative governance in relation to ‘trust and power’ where ‘power holders can use their power to determine formal institutional rules and templates regulating participant’s behaviours as well as to affect informal routines and practices shaping participants perceptions, cognitions and preferences’ [35–37]. We recommend stakeholders and policymakers involved in similar projects to be open and transparent about their own power influence, since this may inhibit innovation and ‘out of the box thinking’.

Leadership was both an important driver as well as important within the CGR. The observations and interviews showed that the informal leader played a major role in inspiring and gathering all the stakeholders, stimulating group empowerment and mediation, and steering the different projects of the ICA, even after an independent chairman had been appointed to chair the daily board and ambassador meetings. The importance of leadership is also evident in literature on collaborative governance [11, 15, 32]. Leadership is a key factor in leading the process, mediating between stakeholders, solving technical problems and managing power imbalances [12, 21, 38].

Huxham et al. describe leadership even more broadly, as something that is not only present in team members, but also present in the system around project members [15]. The situational leadership theory of Hersey et al. [39] describe that leadership should be flexible and that leadership styles evolve over time. In the ICA, we saw that, at the start, the informal leader had a more directive leadership style, and with the transformation of the ICA over time, the leadership style became more coaching and supportive. This connects with the literature on leadership styles, where a persuasive leadership style is preferred above an authoritative leadership style [40, 41].

Although strong leadership is an important aspect of the ICA and within the literature on collaborative governance, it is also described as an inhibitor for broadly carried innovation as stakeholders involved may (unconsciously) follow the vision of this one leader. Uhl-Bien et al. [42] suggest in their research that if leadership involves actively influencing others, then followership involves allowing oneself to be influenced. Shamir et al. [43] attenuate this ‘following’ role of the followers by offering a constructionist sight where the role of the follower is to work with the leader to advance the goals, vision and behaviours essential for both work unit and organisational success. Our suggestion for policymakers who are engaged in collaborative governance is to pay attention to the possible difference in ideas between the ‘leader’ and his/her ‘followers’, even if the leadership is informal.

Overall, having used the Emerson model of collaborative governance we framed the concepts for Principled Engagement, Shared Motivation and Capacity for Joint Action in a different way. Where we speak of goalsetting, transparency, physical presence and informal meetings, we consider these concepts in line with ‘discovery, definition, deliberation and determination as mentioned by Emerson et al. The main concept for Shared Motivation is framed as trust, but comes close Emerson’s concepts of ‘mutual trust, mutual understanding, legitimacy and shared commitment’. The same counts for Capacity for Joint Action, which we framed as leadership and operating protocols, while Emerson et al. frame it as ‘procedural arrangements, leadership, knowledge and resources’ [21]. Due to our case context we preferred to frame it in frequently occurring wording in our context, but overall the collaborative governance model suits well in our case.

Our focus in this study was the starting phase of an ICA. Ulibarri et al. [27] assessed in their study 39 cases of collaborative governance in the starting phase to investigate if there are patterns between the cases. Ulibarri et al. found that the activation phase is a turbulent time with time and context related challenges (for example formulation objectives and goals and single leadership) which is congruent with our findings.

To our knowledge this is one of the first case studies where population health management in four deprived neighbourhoods in Maastricht is analysed with the framework of Emerson et al. We saw that trust emerged as an important variable in the starting phase of this collaborative and affected other variables as well. The complexity of health and social problems faced by the four deprived neighbourhoods may have added to the importance of trust as observed in the starting phase of the ICA. The stakeholders involved were aware from the start that an integrated approach and interorganizational collaboration from a population health management perspective was needed to improve health outcomes in these neighbourhoods.

### Strengths and limitations

We believe the role of the independent researcher and the team is a strength of this study. The researcher created a trustworthy relationship with all stakeholders, which resulted in being able to join confidential meetings. Another strength of the study is the use of a theoretical framework. Since the data is rich and the characteristics are intertwined with each other, the framework helped to analyse the data to meet the requirements of an empirical research study. A limitation of this study may be that the researcher did not join (all) unofficial bilateral meetings between the stakeholders. In these informal meetings, discussions were smoothed out before entering the official meetings. Another limitation is the case study design, since the findings in this study may be context related.

## Conclusion

The objective of this study was to investigate which characteristics of collaborative governance facilitate or hamper collaboration at the start of an integrated community approach aimed at improving population health, quality of care, controlling health care costs and improving professional work satisfaction. We performed a qualitative case study and used observations, interviews and documents.

We found that having shared goals was the foundation of the ICA and having different expectations within these overarching goals was permitted as long as stakeholders were open about these expectations. Trust was the underlying characteristic, which made dialogue about these differences possible. Being physically present at formal and informal meetings stimulated trust among the stakeholders, since this time investment was needed to ‘get to know’ each other and was also perceived as individual and organisation effort for participation in the ICA. We found that the extensive accountability structure in the ICA both hampered (time-consuming, which hindered innovation) and facilitated (kept everybody on board) collaboration. Power may have played a role in trust building and extensive accountability structures; however, this is a characteristic we did not specifically address in our research. In addition, the involvement of a strong (informal) leader who inspired and gathered all the stakeholders, stimulated group empowerment and mediation, and steered the different projects had a major impact on collaborative governance in this ICA.

This case study is of significance for policy, practice and research. This study fills the knowledge gap on the governance of population health management approaches. Policymakers and practitioners can use our lessons learned for implementing similar (population health) initiatives.

From a scientific angle, this study contributes to the evolutionary theory on collaborative governance using the framework of Emerson et al. since trust and power seemed to be additional concepts to the framework in the starting phase of the ICA. We recommend that more scientific research should be conducted into these concepts, looking into the governance of population health management approaches

## Data Availability

The qualitative data that support the findings of this study are available on reasonable request from the corresponding author. The data are not publicly available because they contain sensitive organizational information and information that could compromise research participant privacy. Therefore, raw data will not be provided in a supplementary file or by depositing it in a public repository.

## Abbreviations

CBS: Statistics Netherlands
CEO: Chief Executive Officer
CGR: Collaborative Governance Regime
ICA: Integrated Community Approach
KPI: Key Performance Indicators
NHS: National Health Service
SES: Socioeconomic Status
ZonMw: The Netherlands Organisation for Health Research and Development
